# Overwhelming postsplenectomy infection due to *Mycoplasma pneumoniae *in an asplenic cirrhotic patient: Case report

**DOI:** 10.1186/1471-2334-11-162

**Published:** 2011-06-08

**Authors:** Feng Xu, Chao-Liu Dai, Xing-Mao Wu, Peng Chu

**Affiliations:** 1 Department of Hepatobiliary and Splenic Surgery, Shengjing Hospital, China Medical University, 36, Sanhao Street, Shenyang, Liaoning, P.R.China; 2 Department of intensive Care Unit, Shengjing Hospital, China Medical University, 36, Sanhao Street, Shenyang, Liaoning, P.R.China

**Keywords:** Overwhelming Postsplenectomy Infection, Mycoplasma Pneumoniae, DIC, ARDS

## Abstract

**Background:**

*Mycoplasma pneumoniae *infection is usually self-limited, but some fulminant cases are fatal, even when occurring in previously healthy individuals. It can also be the cause of overwhelming postsplenectomy infection (OPSI).

**Case presentation:**

We report a case of OPSI in a 41-year-old woman with hypersplenism associated with hepatitis B cirrhosis. We detected a significant *Mycoplasma pneumoniae *agglutination titer, but no evidence of infection with *Chlamydia pneumoniae, Legionnella spp*., or any other bacterial or fungal pathogens. She eventually died despite aggressive therapy.

**Conclusions:**

*M. pneumoniae *could be an underestimated cause of OPSI, and should be suspected in fulminant infectious cases in asplenic patients.

## Background

Overwhelming postsplenectomy infection (OPSI) is a rare condition and extremely dangerous in asplenic individuals. The most common pathogen associated with OPSI is *Streptococcus pneumoniae *[1]. There have not been any published case reports on OPSI, in which *Mycoplasma pneumoniae *was implicated. We report here on an adult patient who had undergone splenectomy for hypersplenism associated with hepatitis B cirrhosis and eventually developed OPSI possibly caused by *M. pneumoniae*.

## Case presentation

**Figure 1:**
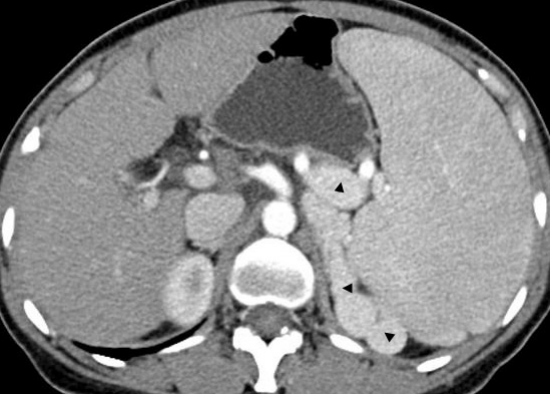
**Contrast computed tomography revealing splenomegaly and varicose veins (triangles)**.

On November 14, 2010, a 41-year-old woman was admitted to our hospital with complaints of high fever and chills of 10-hour duration. Three weeks prior she had undergone a splenectomy for pancytopenia associated with splenomegaly caused by hepatitis B cirrhosis-related portal hypertension without esophageal varices (Figure 1). Her postoperative course was uneventful, and she was discharged 14 days after her operation. Due to her non-compliance, she was not administered the immunoprophylaxis vaccination. Immediately before her onset of fever, she had taken a sponge bath. Acetaminophen did not relieve the fever, and she was admitted to the local hospital 3 hours later when her temperature was 40.5°C. Her physicians only treated the fever and did not administer antibiotics. Three hours later she became disoriented, astatic, and incontinent of urine. At this time she was admitted to our hospital. On initial physical examination, her temperature was 38.2°C; blood pressure, 52/26 mm Hg; pulse rate, 130 beats/min; and respiratory rate, 24 breaths/min. She was conscious. Lip cyanosis, skin petechiae, and ecchymoses were not noted. Her pulmonary examination was unremarkable, and her abdomen had no tenderness or rebound tenderness. Her extremities were cold and no edema was present. Abnormal laboratory findings included the following: white blood cell count (WBC), 11,800/mm^3^; percent neutrophils, 97.6%; lymphocyte count, 200/mm^3^; hemoglobin, 83 g/L; platelet count, 217 × 10^3^/mm^3^; albumin, 22.6 g/L; prothrombin time (PT), 18.9 sec; fibrinogen, 0.9 g/L; D-dimers, 2098 μg/L; pH, 7.091; ABE, -20.9 mmol/L; HCO_3_^-^, 7.2 mmol/L; and pCO_2_, 24.8 mm Hg. Her bedside abdominal ultrasound was unremarkable.

During her admission evaluation, she received intravenous normal saline, plasma, and dopamine (14 μg/kg/min). Her blood pressure increased to 90/62 mm Hg. After she was diagnosed with OPSI and septic shock syndrome, she received intravenous imipenem plus cilastatin 1 g, ulinastatin 100,000 U, and hydrocortisone 100 mg. Sodium bicarbonate was given to alleviate metabolic acidosis. She developed hemodynamic instability 4 hours later, and her blood pressure decreased to 72/31 mm Hg. She was then transferred to intensive care unit, where central venous catheters were introduced for fluid resuscitation, and norepinephrine and dopamine were administered to maintain blood pressure. She received oxygen and was given intravenous vancomycin, linezolid and ornidazole. At first her condition improved, only slightly, but twenty hours after admission, the patient became dyspneic and received noninvasive mechanical ventilation.

**Figure 2:**
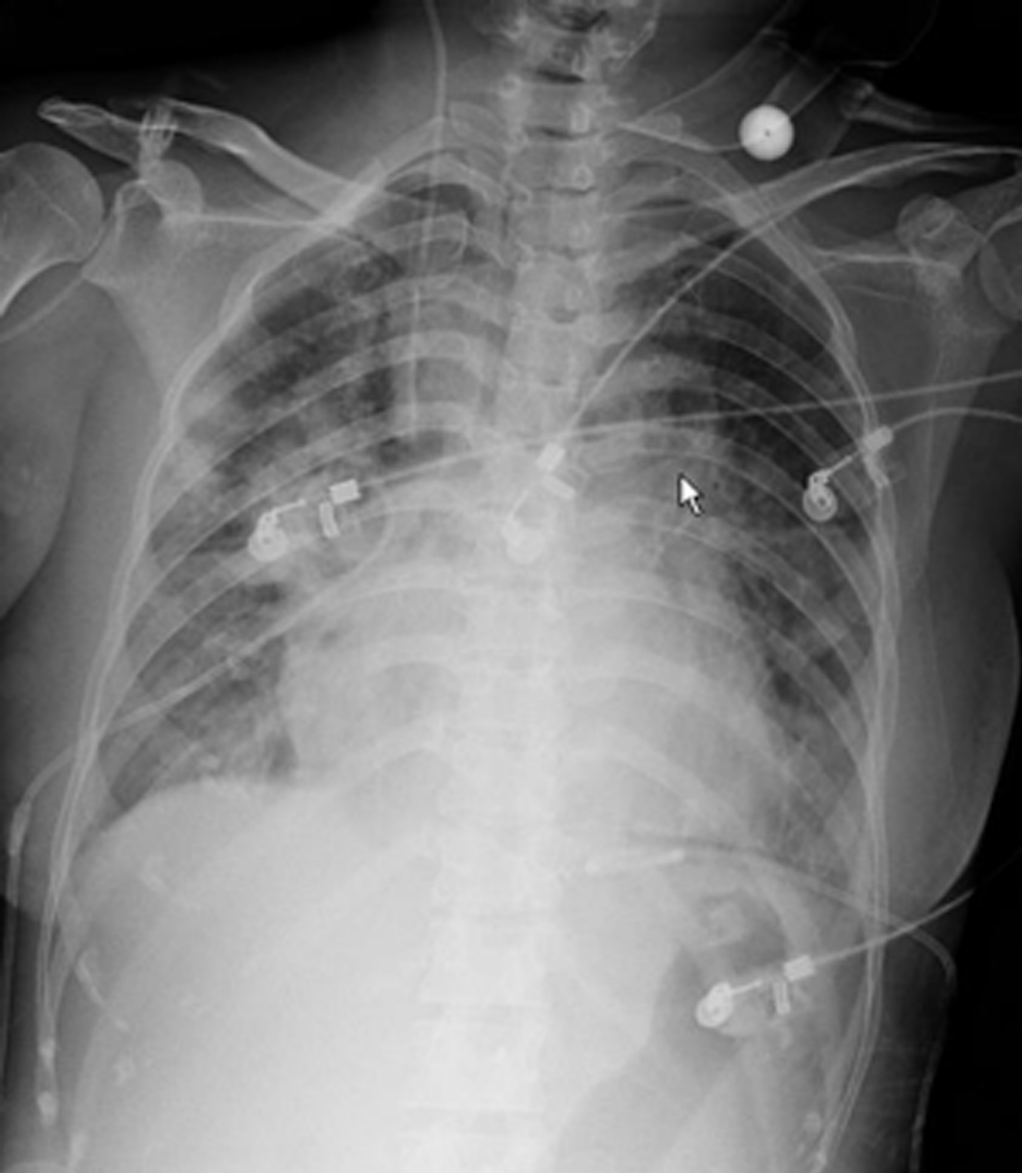
**Chest x-ray showing diffuse infiltrates**.

Results of additional laboratory tests were as follows: WBC, 26,800/mm^3^; PT, 21.7 sec; D-dimers, 3767 μg/L; fibrinogen degradation products (FDP), 181.4 mg/L; troponin I, 18.84 ng/mL; and lactic acid, 11.7 mmol/L. Agglutinating antibodies to *Chlamydia pneumoniae *and *Legionnella spp*. were negative, but significant agglutination titers of 1:640 were seen for *M. pneumoniae*. Bedside chest radiography revealed diffuse bilateral infiltrates, and the bedside ultrasound revealed hydrothorax and ascites (Figure 2). After infection with mycoplasma was established, azithromycin was administered intravenously. Nine hours later, her arterial oxygen saturation decreased to 71%, and an endotracheal tube was inserted to support respiration. Hemodialysis was performed to correct lactic acidosis. Fifty-four hours after admission, despite intensive treatment, she developed progressive hemodynamic deterioration and disseminated intravascular coagulation (DIC). Due to financial concerns, she was discharged without further treatment. She died immediately after returning to her home. An autopsy wasn't preformed due to the husband's request. Cultures of blood, sputum, and drainage fluid were all negative for bacteria and fungi.

## Conclusions

The spleen is the largest organ of the lymphatic system, and is crucial for host response to infection because of its function in removing microorganisms and their products from the circulation. It is also the site of antibody production. So asplenic patients are immunocompromised, with a high rate of morbidity and mortality from fulminant sepsis. 4.4% of children under 16 years of age who receive a splenectomy will develop OPSI, as compared to only 0.9% of adults who have the same procedure [2]. The mortality rate is still 50% to 70% despite improvements in antibiotic therapy and intensive care [1]. In asplenic patients, the increased risk of OPSI is lifelong ordeal. During the first 4 years after splenectomy, 50% to 70% of patients with OPSI were hospitalized [3]. OPSI has a remarkably broad occurrence timetable. It has been reported to occur from 24 days to long as 65 years after a splenectomy [3]. Our patient developed OPSI only 21 days after her splenectomy procedure. In her case, the development of OPSI may have also been associated with hepatitis B cirrhosis, which is manifested by protein and energy malnutrition and reduced host immune response [4,5]. Resecting the spleen caused her immune system to be even more vulnerable to attack, thereby increasing her susceptibility to OPSI.

Sepsis in splenectomized patients can develop after infection with any organism, including bacteria, viruses, fungi, and protozoa; however, encapsulated organisms are most frequently associated with sepsis in asplenic patients. *Streptococcus pneumoniae*, responsible for 50% to 90% of cases, is the most dangerous pathogen. *Haemophilus influenzae *type B, *Neisseria meningitidis*, and group A *Streptococcus *have accounted for additional infections [6]. In our patient, cultures of blood, sputum, and drainage fluid, all collected before antibiotic administration, were negative for bacteria and fungi. Serological testing was strongly positive for agglutinating antibodies to *M. pneumoniae *(1:640), and bedside chest radiography revealed diffuse bilateral infiltrates. Confirmed diagnosis of *M. pneumoniae *is often difficult and is mostly based on serological methods, which require highly acute antibody titers or paired serum samples for definitive diagnosis, since direct detection or culture is time consuming and not readily available [7]. Given this information, while we are unable to obtain definitive evidence of a link, it is our opinion that an acute M. pneumoniae infection was a primary factor in the patient's development of OPSI.

*Mycoplasma *species infection is highly prevalent in the general population, and is usually self-limited. The occurrence of fulminant cases is very rare, primarily occurring in patients with sickle cell trait and related hemoglobinopathies or hypogammaglobulinemia, but it also occurs in previously healthy individuals [8]. An English-language MEDLINE database search on fatal *M. pneumoniae *infections revealed that death was usually due to acute disseminated encephalomyelitis, adult respiratory distress syndrome (ARDS), or vascular thrombosis and DIC, and did not bear any mention of a patient dying from OPSI [7,9,10]. The pathogenic mechanisms involved in these fatal conditions are so far unknown, and most probably involve immune cell-mediated tissue damage. Narita suggested that extra pulmonary manifestations of *M. pneumoniae *infection could be classified into 3 categories. One mechanism involves inflammatory cytokines locally induced by lipoproteins contained in the bacterial cell membrane, while a second involves a form of immune modulation such as autoimmunity resulting from cross-reactivity between bacterial cell components and human cells. Still a third involves vascular occlusion caused by vasculitis and/or thrombosis with or without systemic hypercoagulability, induced by the bacterium [11].

It is well known that treatment of *M. pneumoniae *is effective only when macrolides are administered early in the course of infection. We did not diagnose *Mycoplasma *infection in the patient until one day after she had been admitted to the hospital. It seems that this delay in starting treatment was the primary factor in poor response to high doses of azithromycin. Our patient developed progressive respiratory failure and DIC and eventually died, despite receiving aggressive therapy that included intravenous fluids, antibiotics, vasopressors, steroids, heparin, packed red blood cells, fresh frozen plasma, and continuous hemodiafiltration and plasma exchange.

Prevention of OPSI is paramount in immunocompromised asplenic patients because of its high mortality rate. Recommended preventive strategies include education of patients and relatives, which include, but are not limited to the vaccine immunoprophylaxis against pneumonia, influenza, and meningitis as well as lifelong chemoprophylaxis [12-14]. Asplenic patients should avoid animal and tick bites, and malaria in endemic malaria regions, and should be treated immediately in such settings [12].

Patient education on the potential risks of infection must be reinforced throughout the asplenic patient's life. A previous study has shown that 55.2% of asplenic patients had poor awareness of the risks of splenectomy, and only 1.4% of aware patients developed OPSI compared to 16.5% of patients with poor knowledge of OPSI risks [15]. Therefore, some form of medical alert, such as a bracelet or a card, should be carried by asplenic individuals for constant reminder of their condition and for medical attendants in the event of a medical emergency [13,16]. A spleen registry should also be established to ensure that asplenic patients, family members, and caregivers have easy access to the most current recommendations for the prevention of OPSI [12].

A polysaccharide-based pneumococcal vaccine is recommended for all asplenic individuals, however the timing of vaccination is very important. The optimal time is at least 2 weeks before elective splenectomy about 2 weeks postoperatively for emergency patients, and revaccination every 5-10 years is recommended [12,16]. Studies reveal that no OPSI has been seen in patients vaccinated before splenectomy; however, 5% of patients receiving vaccination after surgery developed OPSI, and 10.4% of patients who were not vaccinated developed OPSI [14,17]. Influenza immunization, in a manner, is more important than pneumococcal immunization [14].

The use of antibiotic prophylaxis remains controversial with some because there currently is no clear clinical data demonstrating its efficacy in asplenic patients, and commonly used antibiotics may result in increased resistance to antimicrobials. However, the Australian Therapeutic Guidelines recommend daily amoxicillin and/or a macrolide for at least 2 years postoperatively [12].

In conclusion, *M. pneumoniae *may be a pathogen involved in OPSI, which is a rapidly progressive condition with a high mortality rate and should be treated aggressively. Extremely important prevention strategies include education, vaccination, and chemoprophylaxis.

## Consent

Written informed consent was obtained from our patient's husband for publication of this case report and any accompanying images. A copy of the written consent is available for review by the Editor-in-Chief of this journal.

## Competing interests

The authors declare that they have no competing interests.

## Authors' contributions

All authors read and approved the final manuscript. FX, CLD and PC: Operating team and postoperative cared; FX, CLD and XMW: involved in patient care in ICU and drafted the manuscript.

## Pre-publication history

The pre-publication history for this paper can be accessed here:

http://www.biomedcentral.com/1471-2334/11/162/prepub
